# Comment on “School-Based Obesity Prevention Intervention in Chilean Children: Effective in Controlling, but not Reducing Obesity”

**DOI:** 10.1155/2015/183528

**Published:** 2015-06-11

**Authors:** Peng Li, Andrew W. Brown, J. Michael Oakes, David B. Allison

**Affiliations:** ^1^Office of Energetics and Nutrition Obesity Research Center, University of Alabama at Birmingham, Birmingham, AL 35294, USA; ^2^Division of Epidemiology and Minnesota Population Center, University of Minnesota, Minneapolis, MN 55454, USA

We read with interest the article “School-Based Obesity Prevention Intervention in Chilean Children: Effective in Controlling, but not Reducing Obesity” [1], hereafter “*the article*.” In* the article* [1], nine schools were randomized into intervention and control groups according to the socioeconomic conditions of the children (a stratified randomization design at the cluster level), resulting in five schools in the intervention condition and four schools in the control. The intervention consisted of training teachers to deliver content on healthy eating and to improve the quality of physical education classes. The primary outcome was change in BMI *Z* score between baseline and follow-up. This is a typical cluster randomized controlled trial (cRCT) in which the inferences are intended to apply at the individual (student) level while randomization is at the cluster (school) level [2, 3]. In cRCTs, the potential lack of independence among individuals in the same cluster, that is, intracluster correlation (ICC), creates special methodological issues in both design and analysis. Any individual level analysis without considering the clustering is invalid [3]. Unfortunately,* the article* [1] ignored the clustering in its sample size estimation and final data analysis, which potentially increased type I and type II error rates and put their conclusions in doubt.

*The article* [1] claims the study as a cluster randomized trial; however, the power and sample size estimation in Section 2.1 completely ignores the facts that (1) the sample size of cRCTs consists of cluster number (*K*) and cluster size (*m*), (2) the power is more dependent on the cluster number than cluster size [2], and (3) the “design effect” is caused by the similarity of individuals in the same cluster [2, 3]. The presentation of study sample sizes described in Section 2.1 is therefore misleading and might confuse readers.

More severely, ignoring the clustering in the final data analyses of cRCTs (as done in* the article* [1]) will cause inflated type I error rates by (1) underestimating the variance of intervention effects and (2) using the extremely magnified degrees of freedom (df) in the hypothesis testing. The fact that clusters are nested within intervention conditions makes the df available to estimate the intervention effects much smaller than the df without nested clusters. For a hypothetical cRCT with *m* persons nested within *K* clusters across *c* experimental conditions, there will be *N* = *c∗K∗m* total persons. Because of the impact of clustering, the df for estimating the between cluster variance is the number of conditions multiplied by the number of clusters minus one, or df = *c∗*(*K* − 1), which is far smaller than *c∗*(*K∗m* − 1), used in* the article* [1]. Considering only nine schools that are involved in the trial, the Kenward-Roger small sample df approximation [4] should also be recommended, which has been implemented in some commercial statistical packages including SAS and R.

Furthermore, in order to improve the transparency and utility of cRCTs, the CONSORT 2010: extension to cluster randomized trials [5], states that the ICC and an indication of its uncertainty are to be reported in describing (1) how the sample size is determined and (2) how clustering is taken into account in the statistical analysis. In addition, the reported ICCs are also helpful for those who may subsequently perform similar (replication) studies.

To evaluate the validity of this study, we conducted a permutation/randomization test for the difference in change of BMI *Z* scores, using the data in Table 2. Although the major conclusion of the study—in which the school-based treatment prevented increase in BMI *Z* scores compared to control schools—does have statistical merit (permutation *P* < 0.03), we suggest that the authors redo their analyses taking the clustering into account and report the unconditional ICC (or constituent variance components) and its confidence interval for better practice.
